# How do we know a treatment is good enough? A survey of non-inferiority trials

**DOI:** 10.1186/s13063-022-06911-8

**Published:** 2022-12-16

**Authors:** Naomi Attard, Nikki Totton, Katie Gillies, Beatriz Goulao

**Affiliations:** 1 Health Services Research Unit, University of Aberdeen, Aberdeen, UK; 2 School of Health and Related Research, University of Sheffield, Sheffield, UK

**Keywords:** Non-inferiority trials, Elicitation methods, Opinion seeking, Margin justification, Evidence-based

## Abstract

**Background:**

Non-inferiority and equivalence trials aim to determine whether a new treatment is good enough (non-inferior) or as good as (equivalent to) another treatment. To inform the decision about non-inferiority or equivalence, a margin is used. We aimed to identify the current methods used to determine non-inferiority or equivalence margins, as well as the main challenges and suggestions from trialists.

**Methods:**

We developed an online questionnaire that included both closed and open-ended questions about methods to elicit non-inferiority or equivalence margins, underlying principles, and challenges and suggestions for improvement. We recruited trialists with experience of determining a margin by contacting corresponding authors for non-inferiority or equivalence trials. We used descriptive statistics and content analysis to identify categories in qualitative data.

**Results:**

We had forty-one responses, all from non-inferiority trials. More than half of the trials were non-pharmacological (*n* = 21, 51%), and the most common primary outcome was clinical (*n* = 29, 71%). The two most used methods to determine the margin were as follows: a review of the evidence base (*n* = 27, 66%) and opinion seeking methods (*n* = 24, 59%). From those using reviews, the majority used systematic reviews or reviews of multiple RCTs to determine the margin (*n* = 17, 63%). From those using opinion seeking methods, the majority involved clinicians with or without other professionals (*n* = 19, 79%). Respondents reported that patients’ opinions on the margin were sought in four trials (16%). Median confidence in overall quality of the margin was 5 out of 7 (maximum confidence); however, around a quarter of the respondents were “completely unconfident” that the margin reflected patient’s views. We identified “stakeholder involvement” as the most common category to determine respondent’s confidence in the quality of the margins and whether it reflected stakeholder’s views. The most common suggestion to improve the definition of margins was “development of methods to involve stakeholders,” and the most common challenge identified was “communication of margins.”

**Conclusions:**

Responders highlighted the need for clearer guidelines on defining a margin, more and better stakeholder involvement in its selection, and better communication tools that enable discussions about non-inferiority trials with stakeholders. Future research should focus on developing best practice recommendations.

**Supplementary Information:**

The online version contains supplementary material available at 10.1186/s13063-022-06911-8.

## Background

Randomised controlled trials (RCTs) are considered the gold-standard method to estimate effectiveness of an intervention [18]. RCTs usually compare two (or more) treatments to identify which treatment is best (referred to as superiority trials) [6]. However, there has been increasing interest in RCTs focused on defining whether a new treatment is no worse than, or in other words, good enough (non-inferiority trials) or as good as (equivalence trials) the usual treatment [7, 20, 25]. These designs are often selected if the new treatment offers an advantage, such as being less expensive or invasive than the usual treatment. Exact numbers of non-inferiority and equivalence RCTs can be difficult to identify due to lack of consistent terminology, but prevalence is increasing [21]. Non-inferiority RCTs are more common than equivalence, but this is hard to quantify due to the interchangeable use of terms. However, a review identified an approximate 80–20% split for non-inferiority and equivalence, respectively [16, 23].

To design a non-inferiority or equivalence trial, a margin or interval needs to be defined that indicates the new treatment is good enough or as good as the usual treatment. This has been called an “irrelevance margin” [16]. The chosen non-inferiority or equivalence margin has a direct impact on a trial’s sample size, as well as on the interpretation of its findings. This is analogous to the clinically important difference used in superiority trials [4]. Contrary to superiority trials, where the clinically important difference is often defined by trialists using established methods with guidance on its reporting [5], there is lack of consensus about the best way to determine the non-inferiority margin [2, 3, 22]. Possible ways to select a non-inferiority or equivalence margin have included opinion seeking methods, multiple types of literature reviews, and published guidelines [2].

There are concerns over current selection of non-inferiority margins: a recent review found that 75% of non-inferiority trials selected margins that were too wide [24] and can lead to inappropriate treatment recommendations [2, 22]. The method to derive these margins is often unclear in published trials [2]. To improve the relevance and acceptability of the margins selected, calls have been made to involve patients in their definition [1]. However, the extent to which they are currently involved in this process is unknown.

Given the uncertainty around how margins are derived, we aimed to survey trialists involved in the design and analysis of non-inferiority or equivalence trials to identify their current process of defining margins. Additionally, we aimed to explore their opinions on the process including the main challenges as well as suggestions for improvement.

## Methods

### Study design and data collection

This was a descriptive cross-sectional research study using an online questionnaire through Google Forms. The survey was live during two weeks in June 2021 with reminders sent to all invitees where possible. The questionnaire (Additional file 1) was designed with close-ended and open-ended questions, totalling 28 questions focusing on:

- Trial characteristics (including 11 questions, namely trial name/registry, collected to confirm the design of the trial, its starting year, and whether it was a unique entry in the survey). Trials without a registry were accepted, as they could be at any stage of the research cycle including before registration.
- Methods to derive the non-inferiority/equivalence margin (including five questions about opinion-seeking methods, review of evidence based, and use of guidelines). These methods were selected as they were the most commonly reported methods in past reviews’ [2]. The survey questions were informed by existing systematic reviews on non-inferiority trials [2, 22] and the Difference ELicitation in TriAls project [4].
- Questions related to confidence of responder’s in the overall quality of the margins, as well as their reflection of different stakeholder’s views were asked with seven-point Likert scales (where 1 reflected “completely unconfident” and 7 reflected “completely confident”).
- Participants were asked, in open questions, to justify their responses related to the confidence questions, and offer insights into challenges of the process of defining a non-inferiority or equivalence margin and suggestions for improvement.

It was requested that the questionnaire was completed according to the number of trials performed or designed, meaning that multiple entries from the same respondent may be possible.

### Participant recruitment

The target population of the study were trialists involved in the design and/or delivery of non-inferiority or equivalence trials with the appropriate level of knowledge about the trial’s determination of margins. For simplicity, we will refer to them as trialists. Potential participants were identified through purposeful sampling by a PubMed search with the term “non-inferiority trial” or “equivalence trial” in the title or abstract of the study published between 2001 and 2021. Titles and abstracts of the results were screened, the corresponding author’s contact details were extracted, and the questionnaire was disseminated via email. A similar procedure was performed for clinicaltrials.gov, where search words “non-inferiority” or “equivalence” were used, and a 3-year limit was established (targeting trials that had recently started and avoiding duplication with PubMed results). Contact emails were extracted and used to disseminate the questionnaire. An initial contact was made to the corresponding author with a note asking the link to be passed onto the most appropriate trial team member. The questionnaire was also disseminated via international clinical trial networks (e.g. Trial Methodology Research Partnership, UKCRC, UKTMN, Health Research Board-Trial Methodology Research Network) and on social media (Twitter) and snowball sampling was performed (by identifying trialists in the researcher’s network and asking them to pass the survey).

Inclusion criteria to take part included the following: being involved in a non-inferiority or equivalence trial, being an adult and accepting to participate in the study.

### Data analysis

We aimed to recruit as many trialists as possible in the period available. Although the questionnaire requested information on the trial, all identifiable data was anonymised before analysis. All the questions were mandatory apart from the opinion-seeking and review of evidence base sections, to mitigate missing data. The quantitative data from the close-ended questions were processed in SPSS [13] and presented using the appropriate descriptive statistics. Trials could be at any stage of the research cycle (including design). The trial’s starting year was collected after the survey was closed according to the trial’s registry if available. Questions that included an “other” option allowed responders to provide an open answer (e.g. type of primary outcome). They were reviewed by one member of the team (NA) and discussed with a second member (BG) and re-categorised accordingly. Traditional content analysis of the open-ended questions was performed (https://journals.sagepub.com/doi/10.1177/1049732305276687). Categories were identified by two researchers independently (NA and BG). The findings were discussed until consensus was reached, and categories were agreed and quantified in terms of frequency of mention.

## Results

### Sample characteristics

Table 1 presents the characteristics of the survey respondents and the trials they reported on. We had 41 responses, and all trials were non-inferiority designs, as indicated in their trial registry. Trial’s starting date varied from 2006 to 2022. Most trials reported had been completed (44.0%), more than half were non-pharmacological (51.2%) and the majority had two treatment arms (90.3%). The most common choice of primary outcome was clinical (70.7%), which includes clinical functional outcomes or outcomes informed by clinicians. The median (percentile 25–percentile 75) sample size of the trials reported in the survey was 438 (300–624). Chief investigators and statisticians, representing the trial team, were the most common responders (representing 43.9% and 46.4% of responders, respectively).

**Table 1:** Trial and responder’s characteristics

		Frequency (%) (*N* = 41)
Role	Chief investigator	18 (43.9%)
	Clinical researcher	3 (7.3%)
	Statistician	19 (46.4%)
	Trial manager	1 (2.4%)
Year	2006–2011	8 (19.5%)
	2012–2017	14 (34.2%)
	2018–2022	17 (41.5%)
	Year not available^a^	2 (4.9%)
Current stage of trial	Design	2 (4.9%)
	Recruitment	13 (31.7%)
	Data collection	3 (7.3%)
	Data analysis and interpretation	1 (2.4%)
	Reporting research findings	3 (7.3%)
	Completed	18 (43.9%)
	Not applicable	1 (2.4%)
Type of intervention	Pharmacological	18 (43.9%)
	Non-pharmacological	21 (51.2%)
	Both	2 (4.9%)
Arms	Two	37(90.3%)
	Three	3 (7.3%)
	Four	1 (2.4%)
Primary outcome^b^	Disease-specific quality of life	1 (2.4%)
	Mortality	1 (2.4%)
	Clinical outcome	29 (70.7%)
	Adverse effect	2 (4.9%)
	Patient reported outcome	8 (19.5%)
	Public health outcome	3 (7.3%)

^a^Two trials were pre-registration status and did not have a starting year

^b^More than one option may have been chosen; clinical outcomes include a grouping of “clinical functional outcomes,” and “other” outcomes classified as clinical by the study team (e.g. symptom resolution reported by clinicians)”

### Methods to select a margin

Most responders were aware of and recommended methods presented in the survey (opinion seeking, review of the evidence base, guidelines, feasibility) (Table 2). When asked to recommend methods independent of whether they used them, the least recommended methods were feasibility of sample size (56%) and opinion seeking (68%), compared with review of the evidence (87%) and guideline recommendation (85%). Two responders did not recommend any of the presented methods: one did not recommend any methods and another recommended using the minimal clinically important difference (MCID) to estimate the non-inferiority margin. The majority of the responders (80.5%) reported that they felt the methods that they used were appropriate for the definition of the margin.

**Table 2:** Elicitation methods and rationale to select a margin. Multiple options could be selected by responders

Elicitation methods	Frequency (%) (*N* = 41)		
	Method/s applied to surveyed trial	Method/s aware of	Method/s to recommend
Opinion seeking	24 (58.5%)	33 (80.5%)	28 (68.3%)
Review of evidence base	28 (68.3%)	40 (97.6%)	36 (87.8%)
Margin recommendation by guidelines	22 (53.7%)	35 (85.4%)	35 (85.4%)
Feasibility of sample size	18 (43.9%)	35 (85.4%)	23 (56.1%)
Other	0 (0%)	0 (0.0%)	2 (4.9%)^**a**^
Underlying principles to select a margin			
A realistic difference given the interventions under evaluation	27 (65.9%)		
A difference which would lead to an achievable sample size	17 (41.5%)		
A difference that would be viewed as important by a relevant stakeholder group	33 (80.5%)		
Preserved effect of new treatment compared with an established minimally clinically important difference or an active comparator	2 (4.9%)		

^**a**^None of the above methods and minimal clinically important difference

The most common underlying principle to define a margin, reported by responders, was that the difference would be viewed as “important by the relevant stakeholders” (80.5%), followed by the difference being “realistic given the intervention under evaluation” (65.9%).

Table 3 presents details about the combination of methods used to define non-inferiority margins. Most participants used a combination of opinion seeking and evidence base methods (with or without additional methods) (*n* = 17, 41.5%). However, a significant proportion used evidence based only (*n* = 11, 26.8%), followed by opinion seeking only (*n* = 7, 17.1%) and neither (using either feasibility rationales, or recommended margins in guidelines; *n* = 6, 14.6%). There were three trials out of eight (37.5%) using opinion seeking (with or without evidence base) before 2012, compared with seven out of 14 from 2012 until 2017 (50%), and 13 out of 17 from 2018 onwards (76.5%).

**Table 3:** The methods used to elicit the margin including the opinion-seeking method, review of evidence base, and guidelines used

		Freq. (%) (*N* = 41)
Methods used		
	Opinion seeking without evidence base	7 (17.1)
	Evidence based without opinion seeking	11 (26.8)
	Opinion seeking and evidence based	17 (41.5)
	Neither opinion seeking nor evidence based	6 (14.6)
Used opinion seeking methods		**24/41 (58.5)**
Opinion seeking stakeholder^ab^	Clinicians only	4 (16.7)
	Patients only	1 (4.2)
	Clinicians and patients	2 (8.3)
	Clinicians, patients and researchers	1 (4.2)
	Clinicians and researchers	12 (50.0)
	Research team	2 (8.3)
	Missing	2 (8.3)
Opinion seeking recruitment method^ab^	Convenience	12 (50.0)
	Relevant mailing lists	4 (16.7)
	Convenience and contacting key experts	4 (16.7)
	Via a patient and public involvement panel	3 (12.5)
	Unclear	1 (4.2)
Opinion seeking method used^ab^	Direct questioning	14 (58.3)
	Delphi approach	1 (4.2)
	Threshold for clinical efficacy	7 (29.2)
	Trade-off/elicitation methods	2 (8.3)
Implementation of the opinion seeking method^ab^	Survey only	6 (25.0)
	Face to face meetings only	10 (42)
	Face to face meetings and focus groups or interviews	5 (20.8)
	Survey, face to face meeting and focus group	1 (4.2)
	Missing	2 (8.3)
Used evidence synthesis methods		**28/41 (68.3)**
Used systematic review of RCT^b^	Yes	9 (32.1)
	No	9 (32.1)
	Not available at the time of design	8 (28.6)
	Missing	2 (7.1)
Evidence used to justify the margin^b^	Systematic review or meta-analysis of all relevant RCTs	8 (28.6)
	Observational studies or non-RCTs	4 (14.3)
	Reviewed multiple RCTs	10 (35.7)
	Evidence from one RCT	2 (7.1)
	Missing	4 (14.3)
Used guidelines^b^		**22/41 (53.7)**
Guidelines used^b^	EMEA 2006	4 (18.2)
	FDA 2010	4 (18.2)
	ICH E10	1 (4.5)
	ICH E9	2 (9.1)
	Missing	11 (50.0)

*RCT*, randomised controlled trial; *EMEA*, European Medicines Agency; *FDA*, Food and Drug Administration; *ICH*, International Council for Harmonisation of Technical Requirements for Pharmaceuticals for Human Use; *SPIRIT*, Standard Protocol Items: Recommendations for Interventional Trials

^a^More than one option may have been chosen

^b^Only those that used this method answered

Of the 24 trials that implemented an opinion seeking method, more than half consulted clinicians with or without other researchers (*n* = 19, 79.2%). Patients, with or without clinicians and researchers, were consulted in four trials out of 24 (16.7%). Two trials consulted only the research team (8.3%). Most trials recruited the elicitation participants based on convenience (*n* = 14, 58.3%). To elicit the non-inferiority margin, more than half of the trials used direct questioning (*n* = 14, 58.3%); other methods included pre-identifying a threshold from the literature to discuss with experts (*n* = 7, 29.2%), or trade-off methods (*n* = 2, 8.3%) and the Delphi method (*n* = 1, 4.2%). A median (percentile 25–percentile 75) of 14 (4–32) individuals was estimated to have participated in the opinion-seeking process, but eight trials did not provide a precise number (33.3%).

Out of the 27 trials using a review of evidence to define a non-inferiority margin, more than half used either a systematic review of appropriate RCTs (*n* = 8, 29.6%) or a review of several RCTs (*n* = 9, 33.3%). For the 22 trials that consulted guidelines to define the non-inferiority margin, European Medicines Agency (EMEA) 2006 (*n* = 4, 18.2%) and US Food and Drug Administration (FDA) 2010 (*n* = 4, 18.2%) were the most commonly referenced guidelines.

### Confidence in the selected margin

**Fig. 1:**
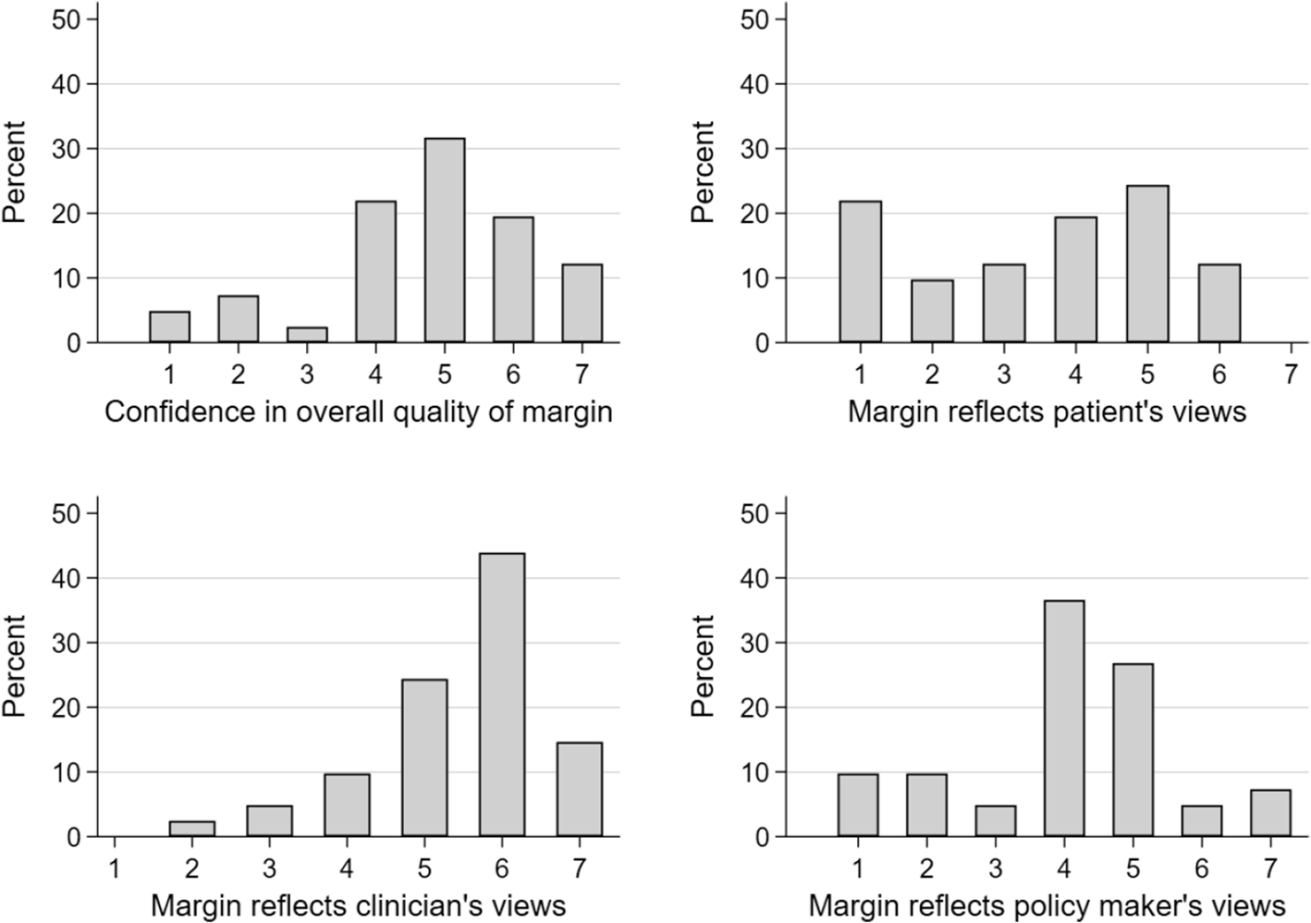
Graphs representing the different levels of confidence of the seven point Likert scale (1—completely unconfident, 7—completely confident) in the overall quality of the margin and whether it represents the views of clinicians, patients and policymakers

As presented in Fig. 1, from the 41 responses, when respondents were asked to score their overall confidence in the quality of the non-inferiority margin most scored as “somewhat confident” (31.7%) with 12.2% of the respondents reporting to be “completely confident” in the margin (median 5.0 out of 7.0, 4.0–6.0). When asked to consider their level of confidence that the margin reflected clinicians’ views, the majority of respondents scored “mostly confident” (43.9%) with no responder selecting completely unconfident (median 6.0, 5.0–6.0). In contrast, 22.0% of respondents were “completely unconfident” the margin represented patients’ views, and the most common response was “somewhat confident” (24.4%) (median 4.0, 2.0–5.0). Policymakers’ views scored highest in the “neither confident nor unconfident” category (36.6%; median 4.0, 3.5–5.0).

Confidence in overall quality of margin differed by the methods used as presented in Table 4, with opinion seeking without evidence base resulting in the lowest average confidence. Confidence in margin reflecting patient’s views differed by group, again with opinion seeking resulting in the lowest average confidence. Confidence in clinician’s views was lowest in the evidence base only group, but similar across the methods. Over 80% of responders considered the method(s) selected appropriate to define their margin in all method groups, except opinion seeking only where this value drops to 57% (*n* = 4).

**Table 4:** Confidence in overall quality and reflection of different stakeholder’s views by method group (Likert scale from 1, completely unconfident, to 7, completely confident)

Method group	Opinion seeking without evidence base (*N* = 7)^a^		Evidence base without opinion seek (*N* = 11)^a^		Opinion seeking and evidence based (*N* = 17)^a^		Neither opinion seek nor evidence base (*N* = 6)^b^	
	Mean (SD)	Median (P25–P75)	Mean (SD)	Median (P25–P75)	Mean (SD)	Median (P25–P75)	Mean (SD)	Median (P25–P75)
Confidence in overall quality	3.9 (1.9)	4.0 (2.0–6.0)	5.1 (1.4)	5.0 (4.0–6.0)	4.8 (1.1)	5.0 (4.0–5.0)	5.2 (2.3)	6.0 (4.0–7.0)
Margin reflects patient’s views	2.7 (2.1)	2.0 (1.0–5.0)	2.8 (1.7)	3.0 (1.0–4.0)	4.1 (1.4)	4.0 (3.0–5.0)	4.2 (1.9)	4.5 (3.0–6.0)
Margin reflects clinician’s views	5.6 (1.3)	6.0 (5.0–6.0)	5.3 (1.1)	5.0 (4.0–6.0)	5.5 (1.3)	6.0 (5.0–6.0)	5.7 (1.0)	6.0 (5.0–6.0)
Margin reflects policy maker’s views	2.7 (1.6)	2.0 (1.0–4.0)	4.4 (1.7)	4.0 (4.0–5.0)	4.0 (1.3)	4.0 (4.0–5.0)	5.2 (1.2)	5.0 (4.0–6.0)
Considered the method appropriate—*n* (%)	4 (57.1)		9 (81.8)		15 (88.2)		5 (83.3)	

*SD*, standard deviation; *P25*, percentile 25th; *P75*, percentile 75th

^a^Methods were used with or without other methods (e.g. feasibility and margin recommendation)

^b^Other methods are feasibility and margin recommendation

### Reasons for assessment of confidence, suggestions for improvement and challenges

Table 5 presents an overview of the categories identified from the open questions with definitions. The most frequent categories identified when justifying overall confidence in non-inferiority margins or confidence in whether margins reflected stakeholder’s views were as follows: “stakeholder involvement” (or lack of) (*n* = 13), “supported by previous literature or guidelines” to inform the margin choice (*n* = 10), and “statistical considerations” (*n* = 7).

**Table 5:** Content analysis of the responses on the questions related to the quality of the non-inferiority/equivalence margin including the theme, definition, and frequency in each theme

Question	Categories	Definition of categories	Freq.
Determinants of confidence in a non-inferiority or equivalence margin quality (*n* = 35)	Stakeholder involvement	Involved or wanted to involve stakeholders in the elicitation	13
	Supported by previous literature or guidelines	Previous literature/guidelines were used in margin design	10
	Statistical considerations	Statistical limitations or comments (e.g. how large the margin is in light of observed event rates)	7
	Feasibility of the trial	Feasibility of the sample size affected the margin	2
	Minimal clinically important difference (MCID)	Non-inferiority margin design was clinically meaningful	2
	Communication	Uncertainty about how clear the communication about the margin was	1
Suggestions to improve the definition of non-inferiority margins (*n* = 33)	Developing methods of involving patients and other stakeholders	Involve different groups/stakeholders in the elicitation process of the non-inferiority margin (e.g. development of formal elicitation methods; sharing of good practice case studies)	14
	More guidance needed	Further guidelines on how to define the margin, including using MCID to inform the non-inferiority margin	10
	Improving methods used	Improve the methods used to justify the non-inferiority margin	4
	Increasing transparency	Make the justification process transparent to others	3
	Pilot or pre-study	Need for pre-studies to determine the non-inferiority margin	2
Challenges in defining non-inferiority margin (*n* = 33)	Communication of non-inferiority trials	Challenges to explain non-inferiority trials to stakeholders	7
	Lack of prior evidence or guidelines	Identifying and using the appropriate guidelines and studies to select the non-inferiority margin	6
	Criteria to determine the feasibility of the trial	Possible constraints that may determine the size or feasibility of the trial	5
	Implementation of results	Assessing whether the margin could inform/persuade clinical practice change	4
	Different views on acceptable margin	Different opinions on what is appropriate for the margin	4
	Lack of method to set the margin or to assess its relevance	Minimal methods available to identify margin and relevance	3
	Variability in outcome literature	Challenges arising from different outcomes or outcome measures being used in different trials, making it harder to take meaningful conclusions regarding non-inferiority margins	3
	Identifying stakeholders	Identifying the appropriate stakeholders to elicit the non-inferiority margin	1

To improve the process of determining the non-inferiority margin, we identified several categories from responders’ suggestions, but the most frequent was developing methods to involve patients and other stakeholders (*n* = 14), followed by “need for better guidance” (*n* = 10).

The most common categories identified related to challenges in defining a non-inferiority margin were: “communication of non-inferiority trials” with stakeholders (*n* = 7), “prior evidence or guidelines” to select the non-inferiority margin (*n* = 6), and “finding criteria to determine the feasibility of the trial” (*n* = 5) (Table 5).

## Discussion

Our survey addresses an important gap in the literature about how non-inferiority margins are being derived in practice: they are crucial to the design and result’s validity of trials, but despite that, over half of the trials published provide poor information on this topic [2, 27]. We collected information from 41 trialists involved in the design and analysis of non-inferiority trials. Most trialists used a combination of evidence base and opinion seeking methods. Trialists had overall confidence on the resulting margin, but this varied depending on the method(s) used. Around a quarter of the sample were “completely unconfident” on the margin reflecting patient’s views. Responders highlighted the need for clearer guidelines on defining a margin, more and better stakeholder involvement in its selection and better communication tools that enable discussions about non-inferiority trials with stakeholders.

Opinion seeking was frequently used to inform the margin selected in the trials surveyed and the method’s usage appeared to increase with time, which is in line with previous research and guidelines [2, 12, 16, 19, 27]. Our results showed that, when using opinion-seeking methods, trialists frequently used convenience samples and direct questioning, which is unlikely to have been assessed for validity (for example, to ensure the question is measuring what it intends to measure) [14]. The precise number of participants involved in the elicitation process was often not known by respondents, which shows the likely informal nature of the process. This adds to previous work that identified the details about the methods used to elicit non-inferiority margins were rarely reported [2, 28] and shows the need for clearer reporting guidance or for better implementation of existing guidance, for example from CONSORT [21]. Recommendations for appropriate methods and reporting requirements could be achieved by building on similar work done for target differences, which included non-inferiority and equivalence margins but did not provide extensive detail [5].

Patients were rarely involved in opinion seeking methods to define non-inferiority margins, and this has since been identified as a top priority for patient and public involvement in numerical aspects of trials [10]. Guidelines recommend that the margin selection should be based on clinical consideration [9]. However, so far, it appears this has mostly been interpreted as the clinician’s views of what is clinically meaningful, and not the patient’s. Patients and clinicians can have different preferences in treatment and different opinions about worthwhile treatment benefits [15, 17], and this should be considered in the design of trials. This finding is line with a recent review of pragmatic clinical trials that found around 4% reported to involve patients or the public in defining the target difference [26].

Responders highlighted that a barrier to achieve stakeholder (including patient) involvement in defining non-inferiority margins was the lack of appropriate methods. Non-inferiority margins are challenging to communicate, and to our knowledge, there is no guidance on how to discuss them with stakeholders in general and patients in particular. Discussing statistical aspects with different stakeholders, including the trial team, has been identified as a challenge [10, 22], which affects patient and public involvement in statistical aspects [8, 11, 22]. Despite this, most respondents (81%) considered ensuring the margin is important by relevant stakeholders as the most important underlying principle to determine it. This is in line with the results obtained in DELTA for underlying principles to determine the target difference in trials [4].

We found that reviewing evidence was frequently used to select a non-inferiority margin, but only a fifth of responders reported using a systematic review of RCTs as recommended by guidelines [7]. Limited use of systematic reviews in the selection of non-inferiority margins could potentially lead to unrealistic margins [22]. However, systematic reviews may not be available when designing a trial. We found a higher percentage of reviewing of evidence base to select a margin when compared to previous reviews. Rehal et al. [22] reported 8% and Althunian et al. [2] reported 11%, but both focused on reported use in published papers, which might be lower than actual use.

Survey respondents highlighted the need to develop guidelines to define non-inferiority margins particularly in a surgical or behavioural intervention context, which is consistent with other studies [2, 22]. This may explain why only half of the respondents reported using guidelines to select the non-inferiority margin, since most relevant guidelines were designed by the pharmaceutical industry.

## Strengths and limitations

We had a relatively small sample size, but we were interested in hearing from a particular group of trialists—those with an adequate level of knowledge about the selection of non-inferiority margins in their trials. This makes the available pool of responders limited. Significant efforts were made to target lead authors of non-inferiority and equivalence trials or roles that might have been involved in defining the margin, including statisticians, producing, for the first time, a more thorough understanding of a complex process. This resulted in a sample of responders that is likely to be particularly knowledgeable about the process of deriving the non-inferiority margin; their views might not be generalisable to other professionals involved in non-inferiority trials. Even though our aim was to capture information for both non-inferiority and equivalence trials, only trialists involved in non-inferiority trials answered the questionnaire. We believe this is likely to be due to a lower number of equivalence trials available; it could also be explained by the fact that non-inferiority and equivalence terms are sometimes, mistakenly, used interchangeably when the primary objective of the trial is to show that an intervention is as effective as the control [22]. In addition, the study team’s contacts, used to snowball the survey, were all from trialists conducting non-inferiority studies. We do not foresee the process of eliciting a margin for an equivalence trial to be different from a non-inferiority trial; therefore, we believe that the results apply to both designs. Our study included a diverse range of trialists and trials, which allowed us to reflect on current practices to determine non-inferiority margins in a variety of settings. The use of mixed methods in this study is innovative in this field, and allowed us to collect richer data and gain a better understanding of the process of defining non-inferiority margins.

## Conclusion

In conclusion, non-inferiority and equivalence margins are crucial to the design and interpretation of trials, but their definition and selection is suboptimal. Trialists in our survey reported using opinion seeking methods and review of evidence base, but the quality of the opinion seeking methods was questionable, and there was uncertainty about the best approach. This uncertainty was also reflected in their confidence about non-inferiority margins reflecting patient’s views. We identified the need for better guidance, as well as methods to involve stakeholders, and tools to improve communication. Future research should focus on developing best practice recommendations, including how to use opinion-seeking methods to determine non-inferiority or equivalence margins and how to communicate about margins to stakeholders and involve them in their selection.

## Supplementary Information

**Additional file 1.** Questionnaire.

## Data Availability

Data is available from the authors upon reasonable request.
