# Supplementary material for: How do we know a treatment is good enough? A survey of non-inferiority trials
Source: Trials. 2022 Dec 16;23:1021. doi: 10.1186/s13063-022-06911-8 (PMC9758907; doi:10.1186/s13063-022-06911-8)
Supplement: Supplementary file 1 — Additional file 1. Questionnaire. [file 13063_2022_6911_MOESM1_ESM.docx]

Additional file 1

**Questionnaire**

***If you have been involved in the design of more than one non-inferiority/equivalence trial, please select one to answer this survey. We suggest, for example, the most recent one if helpful. If you wish to provide information for more than one trial, please complete the survey again.***

1. What is the non-inferiority or equivalence trial name that will be used to complete this questionnaire?

|  |
| --- |

1. If applicable, what is the ISRCTN number of the non-inferiority/equivalence trial?

|  |
| --- |

1. What is/was your role in the non-inferiority/equivalence trial?

Health Economist 🞏
Statistician 🞏
Chief Investigator 🞏
Trial Manager 🞏
Qualitative Researcher 🞏
Clinical Researcher 🞏

1. What is the current stage of the trial?

   Design 🞏
   Recruitment 🞏
   Data collection 🞏
   Data analysis and interpretation 🞏
   Reporting research findings 🞏

Completed 🞏

1. How many arms were included in the trial?

|  |
| --- |

1. Which types of interventions are investigated in the trial? (please tick all that apply)

   Pharmacological 🞏
   Non-pharmacological 🞏
2. What was the primary outcome/s in the trial? (please tick all that apply)

   Generic quality of life 🞏
   Disease-specific quality of life 🞏
   Mortality 🞏
3. Clinical functional measure 🞏
4. Economic outcome 🞏

Adverse effects 🞏
Other: _________________________________________ 🞏

1. What is/was the sample size?

|  |
| --- |

1. Which method/s was used to elicit the non-inferiority and equivalence margins of the sample size? (please tick all that apply)

Opinion seeking (obtain information by asking individuals) 🞏
Review of evidence base (obtain information from previous
studies) 🞏
Margin recommended by disease-specific guidelines 🞏
Feasibility of the sample size 🞏
Other: __________________________________________ 🞏

1. Which methods are you aware of? (please tick all that apply)

Opinion seeking (obtain information by asking individuals) 🞏
Review of evidence base obtain information from previous
studies) 🞏
Margin recommended by disease-specific guidelines 🞏
Feasibility of the sample size 🞏
Not aware of any 🞏

1. Which of the methods would you be happy to recommend?

Opinion seeking (obtain information by asking individuals) 🞏
Review of evidence base obtain information from previous
studies) 🞏
Margin recommended by disease-specific guidelines 🞏
Feasibility of the sample size 🞏
Other: __________________________________________ 🞏

**If in Question 9 you answered “opinion seeking” kindly complete questions 12 to 16.**

1. Who did you seek opinions from to inform the non-inferiority or equivalence margins? (please tick all that apply)

   Clinicians 🞏
   Patients 🞏
   Statisticians 🞏

Pharmaceutical representatives 🞏
Researchers 🞏
Trial team 🞏
Other: __________________________________________ 🞏

1. How did you seek opinions to inform the non-inferiority or equivalence margins? (please tick all that apply)

   Convenience (i.e. trial team and colleagues) 🞏
   Relevant mailing lists 🞏
   Social media 🞏

Conference attendance 🞏
Contacting authors from reference articles 🞏
Word of mouth 🞏
Other: __________________________________________ 🞏

1. How many individuals were consulted in the opinion seeking method?

|  |
| --- |

1. Which opinion seeking method was used to identify the non-inferiority or equivalence margin?

   Direct questioning (for example, asking directly what do you 🞏
   think should be the non-inferiority margin?)
   Bayesian elicitation 🞏
   Delphi Approach 🞏
   Trade-off or preference elicitation methods 🞏

Threshold for clinical efficacy (identified from literature
reviews and discussed with relevant experts to 🞏
establish the non-inferiority margin)
Other consensus approaches: ­______________________ 🞏

Other: __________________________________________ 🞏

1. How did you implement the opinion seeking method? (please tick all that apply)

Survey 🞏
Interview 🞏
Face to face meetings 🞏
Focus group 🞏
Other: __________________________________________ 🞏

**If in Question 9 you answered “review of evidence base” kindly complete questions 17 and 18.**

1. Was the non-inferiority margin based on a Systematic Review of Randomized Controlled Trials?

Yes 🞏
No 🞏
Not available at the time of design 🞏

1. Which evidence was used to justify the non-inferiority/equivalence margin?

   Systematic review or meta-analysis of all relevant 🞏

Randomized Controlled Trials (RCTs)
Reviewed multiple RCTs 🞏
Evidence from one RCT 🞏
Observational studies or non-RCTs 🞏

1. Did you use any guidelines?
   Yes 🞏
   No 🞏
2. If yes, which guidelines were used to design and justify the non-inferiority margin?
   FDA 2010 🞏
   EMEA 2000 🞏
   EMEA 2006 🞏
   ICH E9 🞏

ICH E10 🞏

SPIRIT 🞏

Not applicable 🞏

**Quality of non-inferiority or equivalence margin**

1. (a) How confident were you that the non-inferiority or equivalence margin had good quality?

| Completely unconfident | Mostly unconfident | Somewhat  unconfident | Neither confident nor  unconfident | Somewhat  confident | Mostly confident | Completely confident |
| --- | --- | --- | --- | --- | --- | --- |
| 1 | 2 | 3 | 4 | 5 | 6 | 7 |

(b) Why?

|  |
| --- |

1. (a) How confident were you that the non-inferiority or equivalence margin used reflects patient’s views?

| Completely unconfident | Mostly unconfident | Somewhat  unconfident | Neither confident nor  unconfident | Somewhat  confident | Mostly confident | Completely confident |
| --- | --- | --- | --- | --- | --- | --- |
| 1 | 2 | 3 | 4 | 5 | 6 | 7 |

(b) Why?

|  |
| --- |

1. (a) How confident were you that the non-inferiority or equivalence margin used reflects clinician’s views?

| Completely unconfident | Mostly unconfident | Somewhat  unconfident | Neither confident nor  unconfident | Somewhat  confident | Mostly confident | Completely confident |
| --- | --- | --- | --- | --- | --- | --- |
| 1 | 2 | 3 | 4 | 5 | 6 | 7 |

(b) Why?

|  |
| --- |

1. (a) How confident were you that the non-inferiority or equivalence margin used reflects policy maker’s views?

| Completely unconfident | Mostly unconfident | Somewhat  unconfident | Neither confident nor  unconfident | Somewhat  confident | Mostly confident | Completely confident |
| --- | --- | --- | --- | --- | --- | --- |
| 1 | 2 | 3 | 4 | 5 | 6 | 7 |

(b) Why?

|  |
| --- |

1. Do you think that the method to define the margin was appropriate for the interventions studied?

   Yes 🞏
   No 🞏

1. How can methods to define non-inferiority margins be improved?

|  |
| --- |

1. What do/did you find more challenging about defining a non-inferiority margin?

|  |
| --- |

1. What was the underlying principle(s) adopted in determining the difference (i.e. the non-inferiority or equivalence margin)? (please tick all that apply)

   A realistic difference given the interventions under evaluation 🞏
   A difference which would lead to an achievable sample size 🞏

A difference that would be viewed as important by a relevant 🞏
 stakeholder group
 Other: __________________________________________ 🞏

1. (a) Would you be happy for us to contact you if we have further questions?

   Yes 🞏
   No 🞏

(b) if ‘yes’ please provide contact details:

|  |
| --- |
